# The Catalysis Mechanism of *E. coli* Nitroreductase A, a Candidate for Gene-Directed Prodrug Therapy: Potentiometric and Substrate Specificity Studies

**DOI:** 10.3390/ijms25084413

**Published:** 2024-04-17

**Authors:** Benjaminas Valiauga, Gintautas Bagdžiūnas, Abigail V. Sharrock, David F. Ackerley, Narimantas Čėnas

**Affiliations:** 1 Institute of Biochemistry of Life Sciences Center of Vilnius University, Saulėtekio 7, LT-10257 Vilnius, Lithuania; benjaminas.valiauga@bchi.vu.lt (B.V.); gintautas.bagdziunas@gmc.vu.lt (G.B.); 2 School of Biological Sciences, Victoria University of Wellington, Kelburn Parade, Wellington 6140, New Zealand; abby.sharrock@vuw.ac.nz (A.V.S.); david.ackerley@vuw.ac.nz (D.F.A.)

**Keywords:** nitroreductase A, redox potential, nitroaromatic compounds, quinones, reduction mechanism

## Abstract

*E. coli* nitroreductase A (NfsA) is a candidate for gene-directed prodrug cancer therapy using bioreductively activated nitroaromatic compounds (ArNO_2_). In this work, we determined the standard redox potential of FMN of NfsA to be −215 ± 5 mV at pH 7.0. FMN semiquinone was not formed during 5-deazaflavin-sensitized NfsA photoreduction. This determines the two-electron character of the reduction of ArNO_2_ and quinones (Q). In parallel, we characterized the oxidant specificity of NfsA with an emphasis on its structure. Except for negative outliers nitracrine and SN-36506, the reactivity of ArNO_2_ increases with their electron affinity (single-electron reduction potential, *E*^1^_7_) and is unaffected by their lipophilicity and Van der Waals volume up to 386 Å. The reactivity of quinoidal oxidants is not clearly dependent on *E*^1^_7_, but 2-hydroxy-1,4-naphthoquinones were identified as positive outliers and a number of compounds with diverse structures as negative outliers. 2-Hydroxy-1,4-naphthoquinones are characterized by the most positive reaction activation entropy and the negative outlier tetramethyl-1,4-benzoquinone by the most negative. Computer modelling data showed that the formation of H bonds with Arg15, Arg133, and Ser40, plays a major role in the binding of oxidants to reduced NfsA, while the role of the π–π interaction of their aromatic structures is less significant. Typically, the calculated hydride-transfer distances during ArNO_2_ reduction are smallwer than for Q. This explains the lower reactivity of quinones. Another factor that slows down the reduction is the presence of positively charged aliphatic substituents.

## 1. Introduction

The therapeutic and/or toxic action of nitroaromatic compounds (ArNO_2_) is frequently attributed to the enzymatic reduction of their nitro groups [1,2,3,4,5,6]. In particular, the two-electron reduction of ArNO_2_ leads to formation of nitroso (ArNO) intermediates, which are further reduced into hydroxylamine (ArNHOH) species, which alkylate DNA and other biomolecules. In oxygenated mammalian cells, this is mainly carried out by NAD(P)H:quinone oxidoreductase (NQO1, DT-diaphorase) [7,8,9]. However, because of the low nitroreductase activity of NQO1, a more efficient anticancer conversion of ArNO_2_ can be achieved by gene-directed prodrug therapy (GDEPT) based on the introduction of genes encoding highly active oxygen-insensitive bacterial nitroreductases (NRs) into malignant mammalian cells [10,11]. NRs generally contain flavin mononucleotide (FMN) in the active center and use NAD(P)H as a reducing substrate [12]. Their steady- and pre-steady-state kinetic [13,14,15,16,17,18,19,20,21,22] and crystallographic studies [17,21,22,23,24,25,26] point to a general “ping-pong” mechanism sometimes complicated by substrate inhibition. Their obligatory two-electron reduction of ArNO_2_ and another group of redox active xenobiotics, quinones (Q), may be attributed to an instability of the semiquinone state of flavin [27,28], i.e., to a high redox potential of its semiquinone/hydroquinone couple, which impedes the initial single-electron transfer.

The catalytic activity of NRs towards nitroaromatic compounds is an important parameter that determines the efficiency of GDEPT. For example, low-reduction potential ArNO_2_, which are inefficient substrates for nitroreductases, show low differential cytotoxicity for NR-expressing cancer cells [29]. In this context, *E. coli* nitroreductase A (NfsA) is considered as a candidate for GDEPT because it offers certain advantages over *E. coli* nitroreductase B (NfsB) in the reduction of CB-1954 (5-aziridin-1-yl)-2,4-dinitrobenzamide) and related bioreductively activated bifunctional dinitrobenzenes [11,30,31,32]. Not only has it been shown that NfsA has a ca. ten-fold higher *k_cat_/K_M_* for CB-1954 than NfsB, but NfsA also exclusively reduces the 2-NO_2_ substituent of CB-1954, whereas NfsB can reduce either the 2-NO_2_ or the 4-NO_2_ substituent (but not both) [33]. This has important implications for GDEPT, as reduction at the 2-NO_2_ position yields metabolites that exhibit substantially higher killing of adjacent nitroreductase-null cells. In a solid tumor setting, this “bystander effect” can substantially enhance the efficacy of GDEPT.

Despite the extensive kinetic and crystallographic studies of NfsA [13,20,21,25], there is uncertainty regarding various factors that determine the specificity of substrate reduction. It was found that the reactivity of ArNO_2_ increases proportionate to increases in their electron-accepting potency (defined as a midpoint potential of oxidized compound/anion-radical redox couple, *E*^1^ or *E*^1^_7_ at pH 7.0) and is largely independent of their structural features [20]. However, it remains unclear what factors determine the reactivity of quinones since some of them do not obey the general trend of increasing reactivity with *E*^1^_7_ [20]. Similar observations were made for the class B nitroreductase of *E. cloacae* [15,16]. It was further noted for both enzymes that the reactivity of Q was lower than that of ArNO_2_ with the same *E*^1^_7_ values. These phenomena are atypical for the single-electron or, in some cases, two-electron reduction of these compounds where, as a rule, Q are more reactive than ArNO_2_ [6,8,9,18,34].

In this study, complementing the previous data of Day et al. [21], we characterized the potentiometric parameters of NfsA, which determine the character of the two-electron reduction it performs. Next, by combining kinetic and computer modeling methods, we shed some light on the substrate specificity of NfsA by demonstrating the possibility of different binding characteristics of Q and ArNO_2_. 

## 2. Results

### 2.1. Potentiometric Studies of NfsA

The previous results of potentiometric studies of NfsA do not rule out the possibility of significant formation of FMN semiquinone [21]. This prompted us to reassess the potentiometric characteristics of NfsA by alternative methods. 

The standard redox potential of flavoenzymes (midpoint potential of oxidized/reduced flavin redox couple, *E*^0^ or *E*^0^_7_ at pH 7.0) may be determined using the Haldane relationship, according to which the ratio of the bimolecular rate constants of forward and reverse reactions gives the equilibrium constant of the reaction (*K*) [35,36]. This in turn is related to the difference in the standard redox potential of the reactants (Δ*E*^0^ = 29.5 mV × log *K* for a two-electron transfer). Because the estimation of kinetic parameters of the reverse reaction, the reduction of NADP^+^ by NfsA, is complicated by the absence of appropriate electron donor, we examined the enzyme reactions using the NADP(H) analogue, 3-acetylpyridine adenine dinucleotide phosphate (APADP(H), *E*^0^_7_ = −0.258 V [37]). In this case, *K* is expressed as the ratio of *k*_cat_/*K*_m_ of APADPH oxidation and APADP^+^ reduction. 

**Figure 1:**
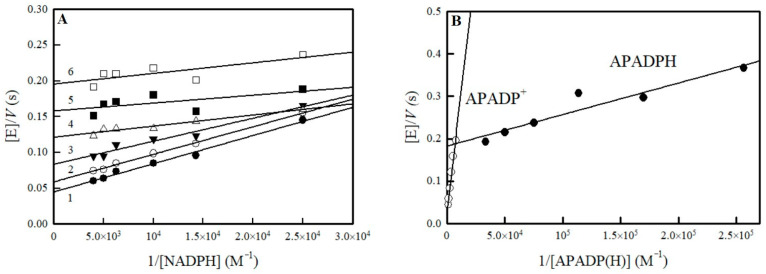
(**A**) Dependence of the rate of NfsA-catalyzed reduction of APADP^+^ on NADPH concentration. Fixed APADP^+^ concentrations: 1.0 mM (1), 0.667 mM (2), 0.444 mM (3), 0.296 mM (4), 0.198 mM (5), and 0.132 mM (6). (**B**) The dependence of maximal rates of APADP^+^ reduction and APADPH oxidation by NfsA on substrate concentration.

APADPH was prepared in situ using a glucose-6-phosphate/glucose-6-phosphate dehydrogenase cofactor regeneration system. The rate of APADPH oxidation by NfsA was monitored using ferricyanide as an electron acceptor because its absorbance did not overlap with that of APADPH. The reaction rate did not depend on the ferricyanide concentration, 0.2–1.0 mM, and was characterized by *k*_cat_ = 5.5 ± 0.6 s^−1^ and *k*_cat_/*K*_m_ = 1.35 ± 0.29 × 10^6^ M^−1^ s^−1^ for APADPH (on the two-electron base). The reverse reaction of NfsA, i.e., reduction of APADP^+^ at the expense of NADPH, proceeds according to a “ping-pong” mechanism, which is complicated by the inhibition by APADP^+^ (Figure 1A). APADP^+^ increases the slopes in the plots 1/[NADPH] vs. [E]/*V*, acting as a competitive inhibitor to NADPH, which reflects its binding to the oxidized enzyme form. Its competitive inhibition constant, *K*_i_ = 150 ± 12 µM, was calculated from the Cleland plots, plotting the dependence of reciprocal *k*_cat_*/K*_m_ of NADPH on APADP concentration. The reduction of APADP^+^ by the reduced NfsA is characterized by *k*_cat_ = 25 ± 4.2 s^−1^ and *k*_cat_/*K*_m_ = 4.3 ± 0.6 × 10^4^ M^−1^ s^−1^. These values were calculated from the dependence of the maximal reaction rates at theoretical infinite NADPH concentrations on the APADP^+^ concentration (Figure 1B). The high ratio of *k*_cat_/*K*_m_ of APADPH and APADP^+^, *K* = 31.4 ± 11.1, gives *E*^0^_7_ of NfsA, −0.215 ± 0.005 V. 

**Figure 2:**
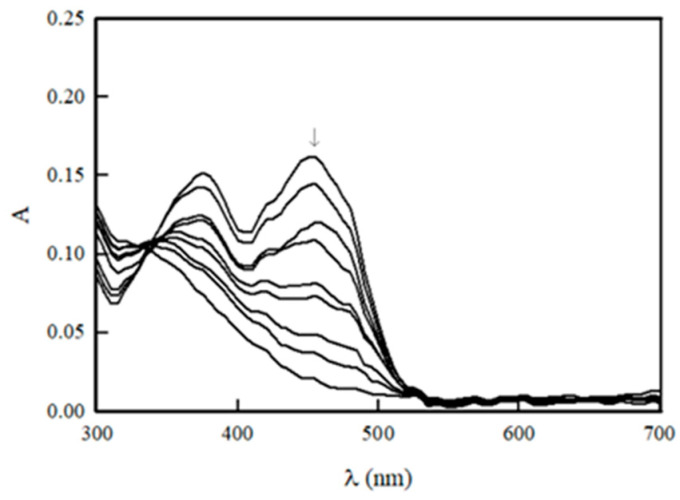
Absorbance spectra of 11.5 µM NfsA during the photoreduction in the presence of 5-deaza-FMN and EDTA. The arrow marks the initial spectrum of NfsA before irradiation, and spectra were recorded after every 10 min of irradiation.

The photosensitized reduction of flavoenzymes by 5-deazaflavins can provide quantitative information on their semiquinone state stability. Compared to the data for *E*. *cloacae* class B NR [27], the extent of photoreduction of NfsA in the presence of 5-deaza-FMN and EDTA reached ca. 90% (Figure 2). 

Analogously, a single isosbestic point in the 336 nm region was observed during the process, indicating that only two redox forms are present during the photoreduction. In addition, we did not observe the noticeable increase of absorbance at 380 nm or 600 nm, which may point to the formation of anionic or neutral FMN semiquinone, respectively.

### 2.2. Studies of NfsA Oxidant Specificity 

**Figure 3:**
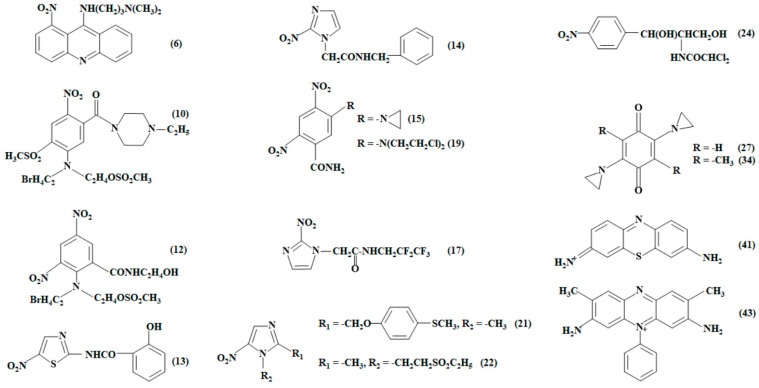
The structures of nontrivial NfsA oxidants tested or discussed in this work. The numbers of compounds correspond to those in Table 1: nitracrine (**6**), SN-36506 **(10**), PR-104A (**12**), tizoxanide (**13**), benznidazole (**14**), CB-1954 (**15**), EF-5 (**17**), SN-23862 (**19**), fexinidazole (**21**), tinidazole (**22**), chloramphenicol (**24**), DZQ (**27**), MeDZQ (**34**), thionine (**41**), and safranine-T (**43**).

Our previous studies of NfsA steady-state kinetics were focused on the relationships between their electron-accepting properties and reactivity [20]. In the current work, we expanded the number of investigated compounds, emphasizing their structural features. These additional compounds include the antiparasitic drugs benznidazole, fexinidazole, and tinidazole; the antiviral agent tizoxanide; the anticancer agents nitracrine, CB-1954, SN-23862, AZQ, and MeDZQ; and the heteroaromatic redox indicators thionine and safranine-T (Figure 3). Other compounds such as anticancer agents SN-36506 and PR-104A, hypoxia PET probe EF-5, and antibacterial agent chloramphenicol (Figure 3) were previously assessed by other teams [31,32,38,39].

The steady-state parameters of reactions, the turnover numbers at infinite oxidant concentration and fixed NADPH concentration (*k*_cat(app.)_), and the bimolecular reduction rate constants of oxidants (or catalytic efficiency constants, *k*_cat_/*K*_m_), which were obtained in the current or previous studies [20,31,32,38,39], are given in Table 1. Because NfsA follows a “ping-pong” mechanism [20], the *k*_cat_/*K*_m_ of oxidants does not depend on the concentration of NADPH. The electron/hydride-accepting characteristics of the compounds used should be mentioned separately. Although NRs reduce ArNO_2_ in a two-electron way, their two-electron (hydride)-accepting potency in aqueous media has not been quantitatively characterized [6]. Therefore, the *E*^1^_7_ of ArNO_2_ was used as a substitute parameter since it parallels to a certain extent the heats of formation of radicals and hydride adducts of ArNO_2_ calculated by quantum mechanical methods [15]. On the other hand, the hydride-accepting potency of quinones and heterocycles in aqueous media is characterized by the hydride-transfer potential (*E*_7_(H^−^)) [40]. *E*_7_(H^−^) is equal to *E*^0^_7_ of quinone if *pK*_a_ of hydroquinone (*pK*_a_(QH_2_) is equal or below 7.0 or the following:

*E*_7_(H^−^) = *E*^0^_7_ − 0.029 V (*pK*_a_(QH_2_) − 7.0 (1)

Table 1 presents *E*^1^_7_ and *E*_7_(H^−^) of compounds, their Van der Waals volumes (VdWvol), and calculated octanol/water partition coefficients at pH 7.0 (log *D*). 

**Figure 4:**
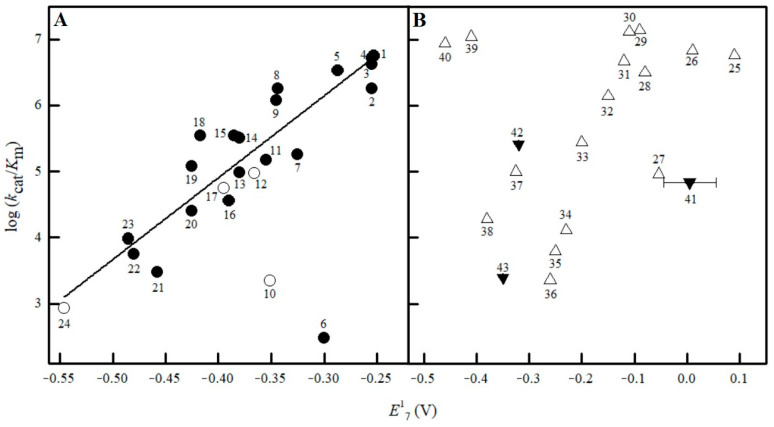
The relationships between the log *k*_cat_/*K*_m_ of nitroaromatics (**A**) and quinones and heteroaromatics (**B**) in NfsA-catalyzed reactions and their *E*^1^_7_. The numbers of compounds correspond to those in Table 1. Nitroaromatics studied in [31,32,38,39] are marked with blank circles; the line corresponds to the first-order regression describing the reactivity of ArNO_2_, except nitracrine (**6**) and SN-36506 (**10**) (**A**). Quinones and heteroaromatics are marked with blank and solid triangles, respectively (**B**).

With the exception of nitracrine (**6**) and SN-36506 (**10**), which were clear negative outliers, the values of log *k*_cat_/*K*_m_ of the tested ArNO_2_ depended linearly on their *E*^1^_7_ (Figure 4A), although the regression was scattered (*r*^2^ = 0.8250, *n* = 22) (Figure 4A). Analogous results were obtained in our previous work with a smaller number of ArNO_2_ (*n =* 12) [20]. Importantly, the log *k*_cat_/*K*_m_ of PR-104A (**12**), EF-5 (**17**), and chloramphenicol (**24**), despite being determined at pH 7.0 in a somewhat different medium [31,32,38,39], correlated well with the data of our previous [20] and present work (Figure 4A). The use of VdWvol as the second correlation parameter did not improve the regression (*r*^2^ = 0.8259). The regression also did not improve using VdWvol and/or VdWvol^2^ as additional variables to detect its optimal value, log *D*, or combinations of these parameters. Q did not possess a well-expressed dependence of log *k*_cat_/*K*_m_ on *E*^1^_7_ (Figure 4B).

Earlier, we identified tetramethyl-1,4-benzoquinone as a negative outlier and 2-hydroxy-1,4-naphthoquinone derivatives as positive outliers [20]. After expanding the number of investigated compounds, we found new negative outliers DZQ (**25**), MeDZQ (**32**), 9,10-anthraquinone-2,6-disulfonate (**33**), thionine (**39**), and safranine-T (**41**) (Figure 4B). Again, their decreased reactivity is not related to their VdWvol since the VdWvol of safranine-T, 333 Å^3^, is close to that of riboflavin, 348 Å^3^ (Table 1, Figure 4B). Since there is some linear dependence between *E*_7_(H^−^) and *E*^1^_7_ values of quinones [40], using *E*_7_(H^−^) as a correlation parameter gives a picture similar to Figure 4B with the same positive and negative outliers.

**Figure 5:**
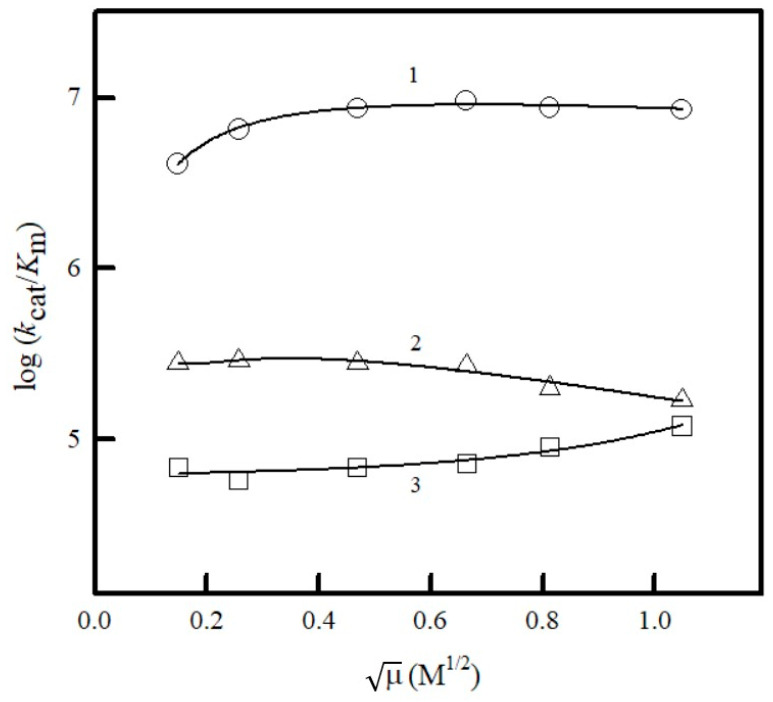
Dependence of log *k*_cat_/*K*_m_ of ferricyanide (1), 2-mehyl-1,4-naphthoquinone (2), and thionine (3) on the ionic strength of phosphate buffer solution.

We investigated the influence of ionic strength on the reactivity of negatively charged ferricyanide, positively charged thionine, and neutral 2-metyl-1,4-naphthoquinone. Interestingly, the reactivity of all these compounds was little affected by the ionic strength of the phosphate buffer solution (Figure 5). 

Next, we investigated the temperature dependence of *k*_cat_/*K*_m_ of several oxidants. The activation enthalpy (ΔH^≠^) of reactions mainly depends on the bond energy change of the reactant system from the initial state to the transition state, whereas the activation entropy (ΔS^≠^) mainly depends on the conformational changes in the formation of the transition state. A more negative ΔS^≠^ indicates that the transition state is more orderly than the initial state of reactants; i.e., greater conformational changes occur during its formation. Our data (Table 2) show that the NfsA-catalyzed reduction of ArNO_2_ is mostly controlled by the activation enthalpy since, in these cases, ΔH^≠^ > −TΔS^≠^. 

Enthalpy control is even more pronounced in the case of 2-hydroxy-1,4-naphthoquinones (Table 2), which are characterized by increased reactivity (Figure 4B). On the other hand, tetramethyl-1,4-benzoquinone, which is a negative outlier (Figure 4B), is characterized by a very negative ΔS^≠^, and its reduction is primarily controlled by the activation entropy (Table 2).

### 2.3. Molecular Modelling of Binding of Oxidants to Reduced NfsA

**Figure 6:**
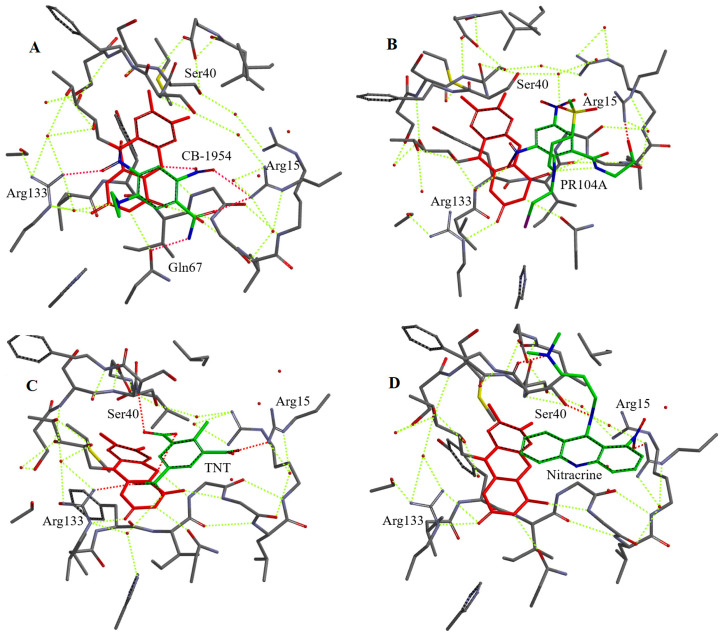
Simplified structures of the active center fragment of NfsA with bound nitroaromatics shown in green: CB-1954 (**A**), PR-104A (**B**), 2,4,6-trinitrotoluene (TNT, (**C**)), and nitracrine (**D**). Isoalloxazine ring of FMN is shown in red, bound compounds are shown in green, H-bonds are shown in dashed lines; water molecules are shown in red dots.

**Figure 7:**
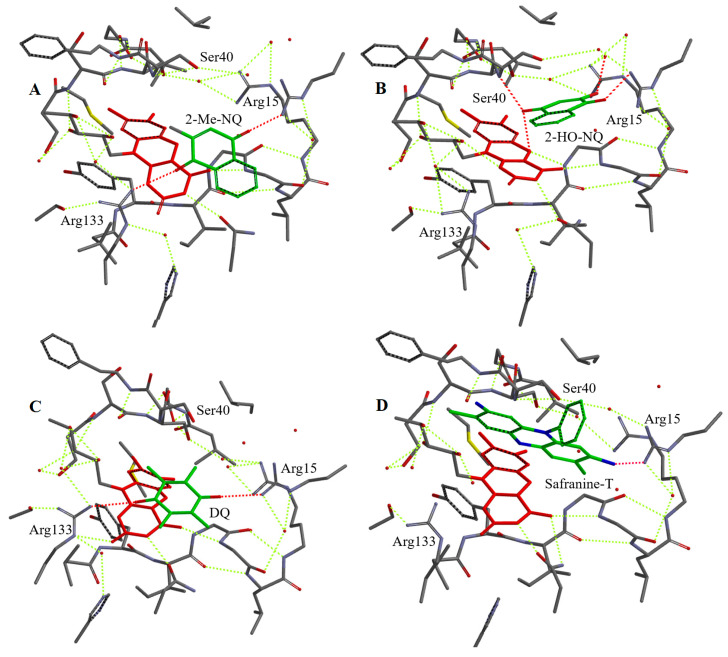
Simplified structures of the active center fragment of NfsA with 2-methyl-1,4-naphthoquinone (2-Me-NQ, (**A**)), 2-hydroxy-1,4-naphthoquinone (2-HO-NQ, (**B**)), tetramethyl-1,4-benzoquinone (DQ, (**C**)), and safranine-T (**D**). Figure designations as in Figure 6.

We selected a number of oxidants of different groups for docking into the active site of NfsA. For this, we used a simplified model involving the enzyme active center fragment around 8 Å from the isoalloxazine ring of FMN, an approach that previously proved successful in predicting the binding modes of various ArNO_2_ in the active center of *Vibrio vulnificus* NfsB [47]. We suggest that, outside this domain, the structure of the enzyme remains unchanged upon the binding of the oxidant. It should be noted that the active center of NfsA contains nine bound H_2_O molecules (Supplementary Figure S1A), which may be displaced upon the binding of the oxidant or may interact with it to form H bonds. In addition, the isoalloxazine ring of FMN was converted into a reduced form for adequate visualization of the reduction pathway. This also prevented possible unproductive orientations of ArNO_2_ towards isoalloxazine, which have been observed in complexes of nitrofurans with oxidized forms of nitroreductases [17,21]. The suitability of our approach is supported by the data of Figure 6A, which shows that the N5 of isoalloxazine is closer to the 2-nitro but not to the 4-nitro group of CB-1954. This is consistent with the preference of NfsA for the reduction of the former group [47]. The structures of the complexes obtained by computer modelling and the calculated distances between the N5 of reduced FMN and hydride-accepting groups are presented in Figure 6A–D, Figure 7A–D, Figures S1B,C and S2A–D (Supplementary Materials), and Table 3. 

All investigated oxidants form H bonds with Arg15 and most of them with Arg133. Thionine and safranine-T do not form H bonds with Arg133 probably due to the specific orientation of their amino groups (Table 1). 2-Hydroxy-1,4-naphthoquinone derivatives bind in a different orientation than other quinones and do not form H-bonds with Arg133 (Figure 7B and Figure S2A), most likely due to the adjacent carbonyl and ionized hydroxyl moiety that can form H bonds with H_2_O and Ser40 (Table 3). The aromatic moieties of the oxidants were found to interact weakly with the C ring of isoalloxazine (Figure 6A–C, Figures S1B,C and S2A–D (Supplementary Materials)), although the calculated plane-to-plane distances for DZQ, 2-methyl-1,4-naphthoquinone, and tetramethyl-1,4-benzoquinone would be favorable for π–π interaction (Table 3). The data of Figure 6D and Table 3 show that the most pronounced negative outlier, nitracrine (Figure 4A), is characterized by the most unproductive binding in the active site of NfsA and the largest distances between the nitro group and N5 of isoalloxazine. 

## 3. Discussion

Class A and B nitroreductases reduce ArNO_2_ and quinones most likely by a single-step hydride (H^−^) transfer [16,19,20]. This may be determined by the low stability of FMN semiquinone at equilibrium [27]. For this reason, we focus primarily on the redox potential of NfsA in the context of other NRs (Table 4) and the stability of its semiquinone.

Our obtained value, *E*^0^_7_ = −0.215 V, does not differ substantially from that determined by Day et al. [21] (Table 4), taking into account a negative shift of redox potential at higher pH and different media composition. However, their results imply that the potentials of transfer of first and second electron are very close, ΔE^1^~0. According to the Nernst equation, this would give about 30% FMN semiquinone at equilibrium. However, in our case, no formation of semiquinone was observed during photoreduction of NfsA (Figure 2). Thus, our data obtained using the alternative method support the view that instability of flavin semiquinone is essential for the two-electron character of oxidant reduction. The complex reaction medium used in the previous study [21] may have influenced the fitting of the electrochemical data. Another conclusion would be that the redox potentials of class A NRs are slightly lower than their class B counterparts (Table 4). This may be one of the factors determining the higher *k*_cat_/*K*_m_ of NfsA compared to NfsB towards low-potential ArNO_2_ such as CB-1954 [11,32]. 

The substrate specificity of NfsA is an important factor for the efficiency of GDEPT, although it is also influenced by other factors—the stability of substrates and their ability to cross various biological barriers [29,50]. When analyzing the NfsA oxidant specificity, it should be noted that besides ArNO_2_, quinones and similar compounds are important not only for studying the mechanisms of NRs but also in a broader aspect: (i) The trypanocidal activity of some quinones is attributed to their reduction by trypanosomal nitroreductases [51], (ii) *Bacillus subtilis* nitroreductases, e.g., YodC, are involved in detoxification of exogenous quinones [52], (iii) low-potential quinones reduced by NfsA and NfsB act as mediators in the bleaching of azo dyes by *E. coli* [53], and (iv) some nitroreductases, e.g., *Enterococcus faecalis* EF0404, also possess azoreductase activity [54].

In this study, we primarily tried to separate the influence of charge transfer energetics (redox potential) and structural features of the compounds in NfsA-catalyzed reactions. Although the linear dependence of log *k*_cat_/*K*_m_ on *E*^1^_7_ is typical for most of the studied ArNO_2_, i.e., their structural features play a minor role, two negative outliers were identified in this work (Figure 4A). We also identified new quinone and heteroaromatic negative outliers (Figure 4B). Since the reactivity of the negative outlier, positively charged thionine, and the model negatively charged oxidant ferricyanide is almost independent of the ionic strength of the solution (Figure 5), this indicates that long-range ionic/electrostatic interactions are not involved here. The H bonds, as short-range interactions, should not be directly affected by ionic strength. 

The obtained results of computer modelling (Figure 6A–D, Figure 7A–D, Figures S1B,C and S2A–D, Supplementary Materials and Table 3) and activation entropy calculations (Table 2) provide indicative information about substrate binding in the NfsA active site. In single-step enzymatic hydride transfer, more negative ΔS^≠^ were attributed to a longer hydride-transfer distance [55] or reduced dynamic mobility of the transition state [56]. Thus, in a simplest case, the hydride-transfer distances should increase in the following order: 2-hydroxy-1,4-naphthoquinones < ArNO_2_ ≤ 2-methyl-1,4-naphthoquinone < tetramethyl-1,4-benzoquinone (Table 2). After excluding the outlier nitracrine, computer modelling data show that the calculated distances from the nitro group of ArNO_2_ to N5 of isoalloxazine are close to those of the carbonyl group of 2-OH-1,4-naphthoquinones (Table 3). In the case of 2-methyl-1,4-naphthoquinone, this distance is larger, but also it is close to those of the outliers tetramethyl-1,4-benzoquinone and DZQ (Table 3). We suggest that the two approaches used give somewhat uncertain results but complement each other. In combination, they suggest that the increased reactivity of ArNO_2_ and 2-OH-1,4-naphthoquinones towards most quinones is associated with a shorter hydride-transfer distance. An increased hydride transfer distance should also be characteristic of negative quinone and heteroaromatic outliers.

Our computer modelling data (Figure 6A–D, Figure 7A–D, Figures S1B,C and S2A–D (Supplementary Materials) and Table 3) show that Arg15, Arg133, and Ser40 may play a major role in substrate binding through H bonds with nitro or carbonyl groups, thereby ensuring their orientation. Therefore, it is important to compare them with the known structural data of NfsA complexes. The data of crystallographic studies show that Ser41, Arg225, and Gln67 form H bonds with the isoalloxazine ring of the bound second FMN molecule, which acts as inhibitor in this case, and with hydroxy groups of bound hydroquinone [21]. The computer modelling shows that Ser 40 and Ser41 could also be involved in the binding of CB-1954 [57] and interact with the 6-nitro group of PR-104A [31]. Other amino acids that could interact with PR-104A are Arg225 (4-nitro group) and Gln67 and His69 (mesylate) [31]. One may note that in these cases, the modelling was performed on the oxidized form of NfsA, in which the non-productive orientation of some compounds is possible [21]. Alternatively, the modelling with reduced NfsA showed that the nitro group of nitrofurantoin could form H bonds with Ser41, Arg225, and Gly131, which ensures a distance of 3.5 Å between the nitro group and N5 of isoalloxazine [21]. We suggest that our data do not contradict those previously obtained because Arg133 is close to Arg225, which forms H bonds with the nitro groups of nitrofurantoin or PR-104A, while Arg15 interacts with the imidazolidine ring of nitrofurantoin [21,31]. Some discrepancies are possible due to the features of the approach we used, as the simulations were performed with the reduced form of FMN, and also, unlike in other studies [31], water molecules were not removed from the NfsA active site. This was carried out on purpose because we have previously shown that water molecules are important for the prediction of supramolecular host–guest structure [58].

Regarding the implications for efficient substrate binding within the active site of NfsA and even of other NRs, our data point to the possible importance of the Arg15 and Ser40 residues. H bonds with these amino acids may be responsible for the productive orientation of 2-hydroxy-1,4-naphthoquinones (Figure 7B and Table 3) and their increased reactivity. Equivalent Ser residues in these positions can be observed in *E. coli* NfsB and the *E. cloacae* B-type NR, where Arg15 is replaced by Lys14 (PDB: 1DSF, 1NEC). The latter enzyme is also characterized by increased reactivity of 2-hydroxy-1,4-naphthoquinones [16]. On the other hand, the low activity of nitracrine towards NfsA may be related to the interaction of its positively charged alkylamine group with Ser40 (Figure 6D and Table 3), its interaction being enhanced by electrostatic repulsion from Arg15. This phenomenon may also be responsible for the diminished reactivity of SN-35606, which has a positively charged *N*-ethylpiperazine group (Figure 3 and Figure 4B). Similarly, analogs of SN-23862 (Figure 3) with positively charged alkylamine substituents at the 1-position were poor or inactive NfsB substrates [59]. The search for more efficient nitroaromatic substrates of NfsA will be carried out in the following stages based on current studies.

## 4. Materials and Methods

### 4.1. Enzymes and Reagents

Recombinant *E. coli* NfsA was purified as described [11,31]. The enzyme concentration was determined spectrophotometrically using ε_460_ = 12.5 mM^−1^ cm^−1^. 2,4,6-Trinitrotoluene and 2-amino-4,6-dinitrotoluene, synthesized as described [9], and DZQ and MeDZQ, synthesized as described [60], were a generous gift of Dr. Jonas Šarlauskas (Institute of Biochemistry, Vilnius, Lithuania). CB-1954 and SN-23682 were synthesized as described [61,62] and were a generous gift of Dr. Vanda Miškinienė (Institute of Biochemistry, Vilnius). Tinidazole, fexinidazole, benznidazole, and tizoxanide were purchased from Selleck Chemicals (Houston, TX, USA); nitracrine was obtained from Polfa (Warsaw, Poland). 5-Deaza-FMN was acquired from Carbosynth Ltd. (Compton, Berkshire, UK). NADPH, 3-acetylpyridine adenine dinucleotide phosphate (APADP^+^), glucose-6-phosphate dehydrogenase, and other compounds were obtained from Sigma-Aldrich (St. Louis, MO, USA). 

### 4.2. Steady-State Kinetic Studies of NfsA

All kinetic experiments were carried out spectrophotometrically using a Perkin Elmer Lambda 25 UV–VIS spectrophotometer (PerkinElmer, Waltham, MA, USA) in 0.1-M K-phosphate buffer (pH 7.0) containing 1-mM EDTA at 25 °C. The steady-state parameters of the reactions, the catalytic constants (*k*_cat(app_._)_), and the bimolecular rate constants (or catalytic efficiency constants, *k*_cat_/*K*_m_) of the oxidants at fixed concentrations of NADPH were obtained by fitting the kinetic data to the parabolic expression using SigmaPlot 2000 (v.11.0, SPSS Inc., Chicago, IL, USA). They correspond to the reciprocal intercepts and slopes of Lineweaver-Burk plots, [E]/*V* vs. 1/(oxidant), respectively, where *V* is the reaction rate, and [E] is the enzyme concentration. *k*_cat(app.)_ represents the number of molecules of NADPH oxidized by a single active center of the enzyme per second at infinite oxidant concentrations. The rates of NfsA-catalyzed NADPH oxidation in the presence of ArNO_2_ and quinones were determined using Δε_340_ = 6.2 mM**^−^**^1^ cm**^−^**^1^. The rates were corrected for the intrinsic NADPH-oxidase activity of the enzyme, determined as 0.10 s**^−^**^1^. For MeDZQ, DZQ, thionine, and nitroaromatic compounds, the reaction rates were additionally corrected for 340 nm absorbance changes due to the reduction of oxidants. For this purpose, the NADPH regeneration system, with 10 mM glucose-6-phosphate and 3 µg/mL glucose-6-phosphate dehydrogenase, was used. The ferricyanide reduction rate was determined using Δε_420_ = 1.03 mM**^−^**^1^ cm**^−^**^1^. The reduction rate of APADP^+^ was determined using Δε_363_ = 5.6 mM**^−^**^1^ cm**^−^**^1^ [62]. APADPH was prepared in situ by the reduction of APADP+ with 10 mM glucose-6-phosphate and 10 µg/mL glucose-6-phosphate dehydrogenase. The APADPH concentration was determined according to Δε_365_ = 7.80 mM**^−^**^1^ cm**^−^**^1^ [37,63]. The enthalpies (ΔH^≠^) and entropies (ΔS^≠^) of activation of reduction of oxidants were calculated from Eyring plots of log (*k*_cat_*/K*_m_)/T vs. 1/T using the data at seven fixed temperatures between 10 and 40 °C. The statistical analysis was performed using Statistica (version 4.3, Statsoft, Toronto, ON, Canada).

### 4.3. Photoreduction of NfsA

NfsA (11–12 µM) photoreduction was performed under anaerobic conditions in 0.10 M phosphate, pH 7.0, using 5-deaza-FMN (0.125 µM) and EDTA (8.0 mM) as the photosensitizers. Before the irradiation, the solution in a Thunberg cell was flushed with O_2_-free argon for 60 min. Subsequently, NfsA was introduced from a concentrated stock solution. The cell was irradiated for short periods at 20 °C with a 100 W incandescent lamp (Osram, Munich, Germany) at a distance of 20 cm; the progress of the reaction was followed spectrophotometrically for 1.0–1.5 h.

### 4.4. Computer Modelling and Calculations 

For computer modelling, the crystal structure of NfsA (PDB ID: 1F5V) was used. To prepare the input files, we first extracted the amino acid residues around 8 Å distance from the N5 atom of the isoalloxazine ring of FMN and generated a minimal structure of the active center using UCSF Chimera software (Version 1.16, University of California, San Francisco, CA, USA). FMN was converted into its reduced form (FMNH_2_). Except the side chains of amino acids capable of forming H bonds with the substrates in the active center, all atoms heavier than hydrogen in these residues were restrained. Additionally, water molecules present in the original crystal structure were allowed to move freely within the active center. The corresponding substrates were placed in the active center, and the obtained structures were relaxed using the molecular mechanics force field (MMFF) method. Subsequently, the structures with and without substrates were optimized using the MMFFaq method, incorporating an aqueous solvent energy correction. This approach enabled us to predict the structure of the complexes and calculate the distances between substrate and enzyme atoms. The Van der Waals volumes of the substrates were calculated from their optimized structures using a hybrid B3LYP functional and a 6-31G(d,p) basis set. All computational analyses were performed using Spartan’18 software (Spartan’18 for Windows Version 1.3.0, Wavefunction Inc., Irvine, CA, USA). 

Octanol/water distribution coefficients at pH 7.0 (log *D*) were calculated using LogD Predictor (https://chemaxon.com (accessed on 2 February 2024)). The statistical analysis was performed using Statistica (version 4.3, Statsoft, Toronto, ON, Canada).

## 5. Conclusions

The potentiometric characteristics of NfsA identified in this study, including the destabilization of the FMN semiquinone, are similar to those of other NRs that catalyze the two-electron reduction of ArNO_2_ and quinones. The determined activation entropies of the reaction, together with the computer modelling data, provided additional information about the factors determining the substrate specificity of NfsA. Although charge-transfer energetics (the redox potential of the oxidant) remains the main driving force for reactions, the reactivity of the substrates can be strongly influenced by their H bonds with Arg15, Arg133, and Ser40. The nature of the interaction with the latter two residues or its absence may influence the mode of binding of the oxidants and confer upon them increased or decreased reactivity. We suggest that our simplified computer modelling approach, focused on the determination of the hydride transfer distance, is a suitable tool for the preliminary selection of potentially efficient substrates for nitroreductases. The selection of appropriate scaffolds has important implications for both GDEPT and other application of NRs.

## Figures and Tables

**Table 1:** Steady-state rate constants of the NfsA-catalyzed reduction of oxidants (*k*_cat(app)_ and *k*_cat_/*K*_m_), their single-electron reduction (*E*^1^_7_) and hydride-transfer (*E*_7_(H^−^)) potentials, Van der Waals volumes (VdWvol), and octanol/water distribution coefficients at pH 7.0 (log *D*). [NADPH] = 100 µM, 0.1 M phosphate + 1.0 mM EDTA, pH 7.0, 25 °C. The values of *E*^1^_7_ are taken from reference [41], and the values of *E*_7_(H^−^) are taken from Reference [40] unless specified otherwise.

No.	Compound	*E*^1^_7_ (V)	*E*_7_(H^−^) (V)	*k*_cat(app.)_ (s^−1^)	*k*_cat_/*K*_m_ (M^−1^s^−1^)	VdWvol (Å^3^)	Log *D*
Nitroaromatic Compounds							
1	2,4,6-Trinitrotoluene	−0.253 ^a^	−	60 ± 5.0	5.6 ± 0.31 × 10^6^	183	2.31
2	*p*-Dinitrobenzene	−0.255	−	71 ± 5.0	1.8 ± 0.10 × 10^6^	142	1.85
3	Nifuroxime	−0.255	−	152 ± 8.0 180 ± 20.0 ^b^	4.2 ± 0.32 × 10^6^ 5.9 ± 0.60 × 10^6 b^	133	−0.34
4	Nitrofurantoin	−0.255	−	119 ± 7.0 136 ± 18.0 ^b^	5.2 ± 0.2 × 10^6^ 7.2 ± 1.0 × 10^6 b^	203	−0.25
5	*o*-Dinitrobenzene	−0.287	−	60 ± 5.5	3.4 ± 0.29 × 10^6^	142	1.85
6	Nitracrine	−0.303	−	−	≤3.0 × 10^2^	336	0.00
7	*p*-Nitrobenzaldehyde	−0.325	−	31 ± 3.0	1.8 ± 0.15 × 10^5^	142	1.63
8	3,5-Dinitrobenzoic acid	−0.344	−	64 ± 5.0	1.8 ± 0.10 × 10^6^	170	−1.79
9	*m*-Dinitrobenzene	−0.345	−	48 ± 5.0 55 ± 6.0 ^b^	1.0 ± 0.08 × 10^6^ 1.2 ± 0.10 × 10^6 b^	142	1.85
10	SN-36506 ^c^	−0.351	−	1.5 ± 0.2	2.2 ± 0.6 × 10^3^	464	−3.40
11	*p*-Nitroacetophenone ^b^	−0.355	−	59 ± 5.0	1.5 ± 0.20 × 10^5^	161	1.47
12	PR-104A ^d^	−0.366	−	8.4 ± 0.6	9.3 × 10^4^	386	0.95
13	Tizoxanide	−0.380 ^a^	−	24 ± 1.8	9.6 ± 0.82 × 10^4^	226	2.16
14	Benznidazole	−0.380 ^a^	−	24 ± 2.1	3.2 ± 0.24 × 10^5^	251	1.32
15	CB-1954	−0.385	−	25 ± 2.0	3.5 ± 0.20 × 10^5^	215	0.64
16	Nitrothiophene	−0.390	−	9.0 ± 0.8	3.6 ± 0.27 × 10^4^	108	1.86
17	EF-5 ^e^	−0.395	−	8.9 ± 0.3	5.6 ± 0.80 × 10^4^	197	1.25
18	2-Amino-4,6-dinitrotoluene	−0.417 ^a^	−	61 ± 4.8	3.5 ± 0.18 × 10^5^	170	1.54
19	SN-23862	−0.425	−	18 ± 1.4	1.2 ± 0.10 × 10^5^	305	2.14
20	*p*-Nitrobenzoic acid ^b^	−0.425	−	64 ± 7.0	2.5 ± 0.20 × 10^4^	149	−1.66
21	Fexinidazole	−0.458 ^a^	−	≥0.6	3.0 ± 0.25 × 10^3^	265	2.38
22	Tinidazole	−0.480	−	2.0 ± 0.3	5.6 ± 0.36 × 10^3^	223	−0.58
23	Nitrobenzene ^b^	−0.485	−	14 ± 2.0	9.6 ± 0.80 × 10^3^	122	1.91
24	Chloramphenicol ^f^	−0.546 ^a^	−	0.89 ± 0.03	8.6 ± 0.90 × 10^2^	270	1.10
Quinones							
25	1,4-Benzoquinone ^b^	0.090	0.195	62 ± 6.0	5.8 ± 0.60 × 10^6^	109	1.02
26	2-Methyl-1,4-benzoquinone ^b^	0.010	0.120	54 ± 6.0	6.8 ± 0.70 × 10^6^	128	1.42
27	DZQ	−0.054	0.000	38 ± 3.0	9.2 ± 0.50 × 10^4^	194	0.0
28	2,6-Dimethyl-1,4-benzoquinone ^b^	−0.080	0.058	51 ± 6.0	3.2 ± 0.40 × 10^6^	146	1.82
29	5-Hydroxy-1,4-naphthoquinone ^b^	−0.090	−0.060	22 ± 3.0	1.4 ± 0.20 × 10^7^	166	1.38
30	5,8-Dihydroxy-1,4-naphthoquinone ^b^	−0.110	−0.084	12 ± 2.0	1.3 ± 0.10 × 10^7^	171	2.19
31	9,10-Phenanthrene quinone ^b^	−0.120	−0.034	54 ± 6.0	4.7 ± 0.50 × 10^6^	212	2.92
32	1,4-Naphthoquinone ^b^	−0.150	−0.029	46 ± 5.0	1.4 ± 0.20 × 10^6^	161	1.49
33	2-Methyl-1,4-naphthoquinone ^b^	−0.200	−0.114	36 ± 4.0	2.8 ± 0.30 × 10^5^	179	1.89
34	MeDZQ	−0.230	−0.128	2.9 ± 0.2	1.3 ± 0.10 × 10^4^	229	0.79
35	9,10-Anthraquinone-2,6-disulfonate	−0.250	−0.210	≥0.7	6.2 ± 0.35 × 10^3^	287	−3.47
36	Tetramethyl-1,4-benzoquinone ^b^	−0.260	−0.086	2.5 ± 0.3	2.3 ± 0.20 × 10^3^	181	2.61
37	1,8-Dihydroxy-9,10-anthraquinone ^b^	−0.325	−	0.7 ± 0.1	1.0 ± 0.15 × 10^5^	224	3.61
38	9,10-Anthraquinone-2-sulfonate ^b^	−0.380	−0.250	1.4 ± 0.1	1.9 ± 0.20 × 10^4^	246	−0.27
39	2-Hydroxy-1,4-naphthoquinone ^b^	−0.410	−0.200	72 ± 8.0	1.1 ± 0.10 × 10^7^	166	−0.99
40	2-Methyl-3-hydroxy-1,4-naphthoquinone ^b^	−0.460	−0.240	56 ± 5.0	8.8 ± 1.50 × 10^6^	184	−0.69
Miscellaneous							
41	Thionine	0.055 ÷ −0.045 ^g^	0.064 ^h^	56 ± 5.0	6.9 ± 0.50 × 10^4^	219	0.8
42	Riboflavin ^b^	−0.320	−0.210	4.2 ± 0.6	2.6 ± 0.20 × 10^5^	348	−1.18
43	Safranine-T	−0.350 ^i^	−0.286 ^h^	≥0.3	2.5 ± 0.21 × 10^3^	333	n.d.
44	Ferricyanide ^j^	0.410		89 ± 6.0	8.6 ± 1.40 × 10^6^	n.d.	n.d.

^a^ *E*^1^_7_ values taken from reference [6]. ^b^ Rate constants taken from reference [20]. ^c^ Taken from references [38,42]. ^d^ Taken from references [30,31]. ^e^ Taken from references [32,43]. ^f^ Rate constants taken from reference [39]. ^g^ Taken from reference [44]. ^h^ Taken from reference [45]. ^i^ Taken from reference [46]. ^j^ Calculated on a single-electron base. The reactions were performed in 0.01 M Tris, pH 7.0 [31,32,38,39]. n.d.—not determined.

**Table 2:** The activation enthalpies (ΔH^≠^) and entropies (ΔS^≠^) of NfsA-catalyzed reactions.

Oxidant	ΔH^≠^ (kJ mol^−1^)	ΔS^≠^ (J mol^−1^ K^−1^)	−TΔS^≠^ (kJ mol^−1^)
Nitroaromatic Compounds			
2,4,6-Trinitrotoluene	24.2 ± 1.7	−34.2 ± 5.7	10.2 ± 1.7
*p*-Nitrobenzaldehyde	27.2 ± 0.9	−55.2 ± 3.1	16.4 ± 0.9
*o*-Dinitrobenzene	23.5 ± 1.4	−33.6 ± 5.2	10.0 ± 1.5
Quinones			
2-Methyl-3-Hydroxy-1,4-naphthoquinone	38.7 ± 1.1	9.7 ± 3.7	-2.9 ± 1.1
2-Hydroxy-1,4-naphthoquinone	28.3 ± 2.1	−16.0 ± 7.0	4.8 ± 2.1
2-Methyl-1,4-naphthoquinone	23.4 ± 1.8	−54.4 ± 6.0	16.2 ± 1.8
Tetramethyl-1,4-benzoquinone	16.4 ± 1.4	−123.0 ± 4.5	36.7 ± 1.3

**Table 3:** Characteristics of NfsA complexes with oxidants calculated by computer modelling.

Compound	H Bonds	Shortest Plane-to-Plane Distance to Isoalloxazine (Å)	Distance to N5 of Isoalloxazine (Å)
Nitroaromatic Compounds			
CB-1954	2-NO_2_…Arg133, 4-NO_2_…Arg15, -CONH_2_…Arg133, Gln67	3.7	2-NO_2_: 2.9 (O), 3.9 (N)
PR-104A	-C_2_H_4_OH…Arg15, 6-NO_2_…H_2_O, -OSO_2_CH_3_…H_2_O	3.8	4-NO_2_: 3.8 (O), 3.4 (N)
2,4,6-Trinitrotoluene	2-NO_2_…Arg15, 4-NO_2_…Arg133, 6-NO_2_…Ser40.	4.0	2-NO_2_: 3.0 (O), 3.4 (N)
*o*-Dinitrobenzene	1-NO_2_…Arg15, 2-NO_2_…H_2_O	3.9	1-NO_2_: 3.0 (O), 3.9 (N)
*p*-Nitrobenzaldehyde	-NO_2_…Arg15, -CHO…Arg133	4.6	3.9 (O), 4.6 (N)
Nitracrine	-NO_2_…Arg15, -N(CH_3_)_2_…Ser40	7.2	8.1 (O), 9.0 (N)
Quinones and Heteroaromatics			
2-Hydroxy-1,4-naphthoquinone	1-C=O…Arg15, 4-C=O…Ser40, 2-O^−^ …H_2_O	3.8	4-C=O: 3.0 (O), 3.5 (C)
2-Methyl-3-hydroxy-1,4-naphthoquinone	1-C=O…Ser40, 4-C=O…Arg15, 3-O^−^…H_2_O	4.2	1-C=O: 3.1 (O), 3.6 (C)
2-Methyl-1,4-naphthoquinone	1-C=O…Arg133, 4-C=O…Arg15	3.3	1-C=O: 5.1 (O), 4.9 (C)
Tetramethyl-1,4-benzoquinone	1-C=O…Arg133, 4-C=O…Arg15	3.3	1-C=O: 5.1 (O), 4.7 (C)
DZQ	1-C=O…Arg133, 4-C=O…Arg15	3.3	1-C=O: 4.1 (O), 4.1 (C)
9,10-Anthraquinone-2,6-disulfonate	2-SO_3_^−^…Arg15, 6-SO_3_^−^…Arg133	4.5	10-C=O: 6.0 (O), 6.3 (C)
Thionine	3(7)-NH_2_…Arg15	3.6	5.4 (S), 5.3 (N10)
Safranine-T	3(7)-NH_2_…Arg15	4.5	5.3 (N5(10))

**Table 4:** Standard redox potentials of class A and B nitroreductases and their homologs NAD(P)H:FMN oxidoreductases.

Enzyme	Standard Redox Potential	Conditions
*E. cloacae* NR (B) [27]	−0.190 V	Equilibration with redox mediator, PIPES, KCl, pH 7.0
*Vibrio fischeri* NAD(P)H: FMN oxidoreductase (B) [48]	−0.215 V	Equilibration with redox mediator, phosphate, pH 7.0
*E. coli* NfsB [49]	−0.199 V	Equilibration with redox mediator, Tris, NaCl, pH 7.0
*E. coli* NfsB [22]	−0.218 V	Direct electrochemical reduction, phosphate, KCl, 10% glycerol, pH 7.5
*Vibrio harveyi* NADPH: FMN oxidoreductase (A) [48]	−0.255 V	Equilibration with redox mediator, phosphate, pH 7.0
*E. coli* NfsA [21]	−0.264 V FMN_ox/sq_: −0.268 V FMN_sq/red_: −0.272 V ^a^	Direct electrochemical reduction, phosphate, KCl, 10% glycerol, pH 7.5
*E. coli* NfsA, this work	−0.215 V	Reactions with APADP^+^/APADPH, phosphate, pH 7.0

^a^ The data fit to two single-electron transfer steps [21].

## Data Availability

The data may be available at corresponding author upon a reasonable request.

## Supplementary Materials

The following supporting information can be downloaded at: https://www.mdpi.com/article/10.3390/ijms25084413/s1.

## Abbreviations

APADP(H)	3-acetylpyridine adenine dinucleotide phosphate
ArNHOH	Hydroxylamine
ArNO	Nitroso compound
ArNO_2_	Nitroaromatic compound
*E* ^0^_7_	Midpoint potential of oxidized/reduced flavin redox couple
*E* ^1^_7_	Single-electron reduction potential
*E*_7_(H^−^)	Hydride-transfer potential
FMN	Flavin mononucleotide
GDEPT	Gene-directed prodrug therapy
*k*_cat_	Catalytic constant
*k*_cat_/*K*_m_	Bimolecular rate constant
*K*_i_	Inhibition constant
log *D*	Octanol/water distribution coefficient at pH 7.0
MMFF	Molecular mechanics force field
NfsA	*Escherichia coli* nitroreductase A
NfsB	*Escherichia coli* nitroreductase B
NR	Nitroreductase
Q	Quinone
VdWvol	Van der Waals volume
ΔH^≠^	Activation enthalpy
ΔS^≠^	Activation entropy
