# Supplementary material for: The Catalysis Mechanism of E. coli Nitroreductase A, a Candidate for Gene-Directed Prodrug Therapy: Potentiometric and Substrate Specificity Studies
Source: Int J Mol Sci. 2024 Apr 17;25(8):4413. doi: 10.3390/ijms25084413 (PMC11049802; doi:10.3390/ijms25084413)
Supplement: Supplementary file 1 [file ijms-25-04413-s001.zip › ijms-2925814-supplementary.pdf]

The Catalysis Mechanism of *E. coli* Nitroreductase A, a Candidate for Gene-Directed Prodrug Therapy: Potentiometric and Substrate Specificity Studies

Benjaminas Valiauga [1], Gintautas Bagdžiūnas [1], Abigail V. Sharrock [2], David F. Ackerley [2], and Narimantas Čėnas [1,*]

[1] Institute of Biochemistry of Vilnius University, Saulėtekio 7, LT-10257 Vilnius, Lithuania; benjaminas. valiauga@bchi.vu.lt (B.V.); gintautas.bagdziunas@gmc.vu.lt (G.B.);
[2] Victoria University of Wellington, School of Biological Sciences, Kelburn Parade, Wellington 6140, New Zealand; abby.sharrock@vuw.ac.nz. (A.V.S.); david.ackerley@vuw.ac.nz (D.F.A.);
* Correspondence: narimantas.cenas@bchi.vu.lt; Tel.: +37052234392;

Supplement S1

Data of computer modelling of the binding of nitroaromatic compounds, quinones and heteroaromatic compounds in the NfsA active site

For computer modelling, the crystal structure of NfsA (PDB ID: 1F5V) was used. To prepare the input files, we first extracted the amino acid residues around 8 Å distance from the N5 atom of the isoalloxazine ring of FMN, and generated a minimal structure of the active center using UCSF Chimera software. FMN was converted into its reduced form. Except the side chains of amino acids capable of forming H-bonds with the substrates in the active center, all atoms heavier than hydrogen in these residues were restrained. Additionally, water molecules present in the original crystal structure were allowed to move freely within the active center. The corresponding substrates were placed in the active center, and the obtained structures were relaxed using the molecular mechanics force field (MMFF) method. Subsequently, the structures with and without substrates were optimized using the MMFFaq method, incorporating an aqueous solvent energy correction. This approach enabled us to predict the structure of the complexes and calculate the distances between substrate and enzyme atoms. All computational analyses were performed using Spartan’18 software (Spartan’18 for Windows Version 1.3.0, Wavefunction Inc., Irvine, CA, USA).

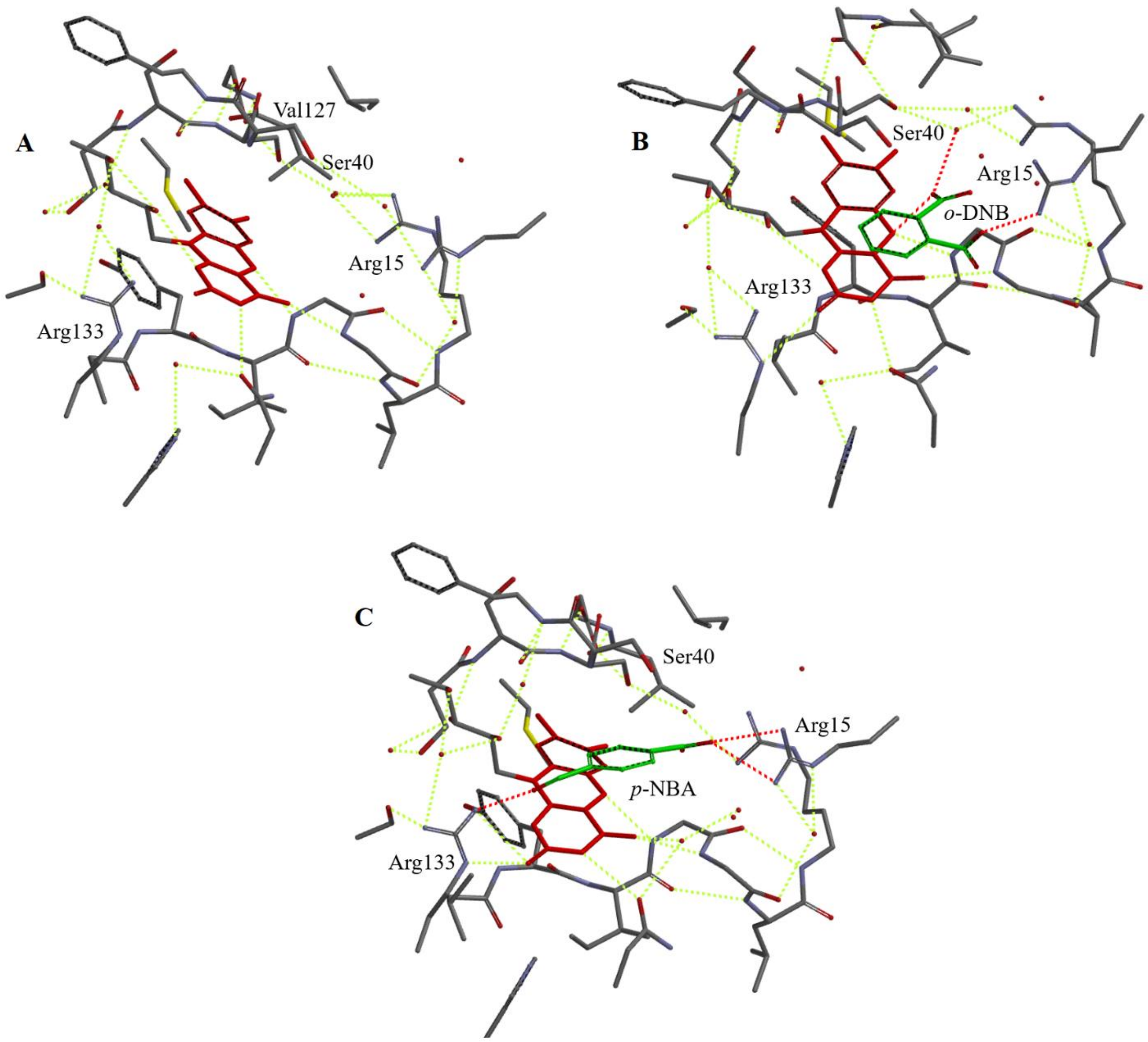

Figure S1. Simplified structures of the active center fragment of NfsA (A) and its complexes with *o*-dinitrobenzene (*o*-DNB) (B) and *p*-nitrobenzaldehyde (*p*-NBA) (C). Nitroaromatic compounds are shown in green, isoalloxazine ring of FMN is shown in red, water molecules are shown in red dots.

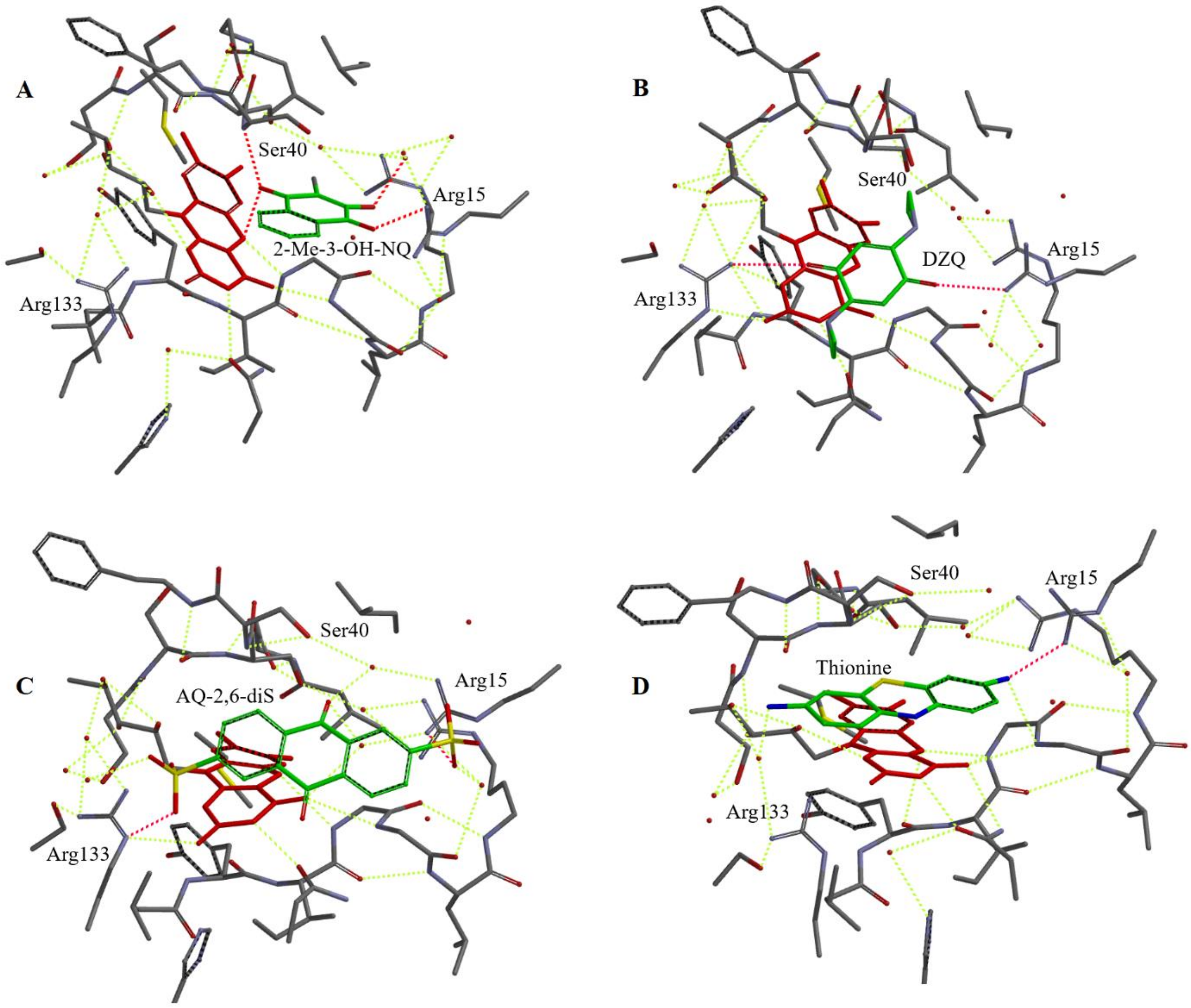

Figure S2. Simplified structures of the complexes of active center fragment of NfsA with 2-methyl-3-hydroxy-1,4-naphthoquinone (2-Me-3-OH-NQ) (A), DZQ (B), 9,10-anthraquinone-2,6-disulfonate (AQ-2,6-diS) (C), and thionine (D). The bound compounds are shown in green, isoalloxazine ring of FMN is shown in red, water molecules are shown in red dots.
